# Vitamin D_5_ in *Arabidopsis thaliana*

**DOI:** 10.1038/s41598-018-34775-z

**Published:** 2018-11-05

**Authors:** Daniele Silvestro, Claire Villette, Julien Delecolle, Carl Erik Olsen, Mohammed Saddik Motawia, Philippe Geoffroy, Michel Miesch, Poul Erik Jensen, Dimitri Heintz, Hubert Schaller

**Affiliations:** 1 Copenhagen Plant Science Centre, Department of Plant and Environmental Sciences, University of Copenhagen, Thorvaldsensvej 40, DK-1871 Frederiksberg C, Copenhagen Denmark; 2 Institut de Biologie Moléculaire des Plantes du CNRS, Université de Strasbourg, 12 rue du Général Zimmer, F–67083 Strasbourg, France; 3 Institut de Chimie, Université de Strasbourg, 1 rue Blaise Pascal, F-67008 Strasbourg, France; 4 Present Address: Carlsberg Research Laboratory, Copenhagen, Denmark

## Abstract

Vitamin D_3_ is a secosterol hormone critical for bone growth and calcium homeostasis, produced in vertebrate skin by photolytic conversion of the cholesterol biosynthetic intermediate provitamin D_3_. Insufficient levels of vitamin D_3_ especially in the case of low solar UV-B irradiation is often compensated by an intake of a dietary source of vitamin D_3_ of animal origin. Small amounts of vitamin D_3_ were described in a few plant species and considered as a peculiar feature of their phytochemical diversity. In this report we show the presence of vitamin D_5_ in the model plant *Arabidopsis thaliana*. This plant secosterol is a UV-B mediated derivative of provitamin D_5_, the precursor of sitosterol. The present work will allow a further survey of vitamin D distribution in plant species.

## Introduction

Here, we report the presence of vitamin D_5_ in *Arabidopsis thaliana*. This species is the most investigated plant as a model in biology however a large part of its genome is still annotated as unknown and its metabolome is not exhaustively described. Steroids represent a diverse group of compounds. Their biogenesis requires the common enzymes of the mevalonate pathway leading to the production of the committed precursor squalene. Post-squalene biosynthetic pathways are implied in the production of cholestane, ergostane and stigmastane classes of sterols, and of downstream metabolites such as cardenolides, steroidal saponins and alkaloids, phytoecdysteroids, or brassinosteroids^1^. Structural elucidation of low abundance metabolites and their functional analysis are often experimentally unrelated. A prominent example is the seminal description of brassinolide in 1979 in rape pollen^2^ prior to the demonstration of its key role in growth and development in 1996 thanks to the power of *Arabidopsis thaliana* forward genetics^3^. In this study, the detection of vitamin D_5_ (or sitocalciferol) in *Arabidopsis thaliana dwarf 5* mutant opens up a new perspective for the production of this overlooked land plant-derived stigmastane-type secosteroid.

**Figure 1:**
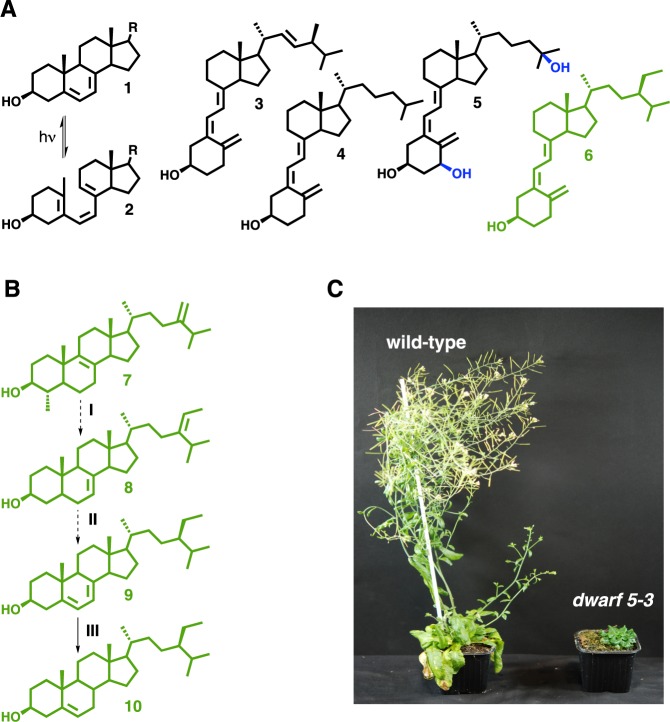
Δ^5,7^-sterols and vitamin D. (**A**) Provitamin D (**1**) is a sterol possessing a conjugated 5,7-dienic system in ring B of the tetracyclic moiety and a side chain R of 8, 9 or 10 carbon atoms defining cholestane, ergostane, or stigmastane series of compounds. UV-B irradiation of fungi or human skin leads to the photoconversion of Δ^5,7^-sterols (**1**) into previtamin D (**2**) stabilized as vitamin D compounds like vitamin D_2_ (ergocalciferol) (**3**) or vitamin D_3_ (cholecalciferol) (**4**), respectively. Vitamin D are metabolized in the body into biologically active forms like 1,25-dihydroxyvitamin D_3_ (**5**). Vitamin D_5_ (or sitocalciferol) (**6**) is a synthetic product from the stigmastane series of compounds. (**B**) Simplified plant sterol biosynthetic pathway showing on ring B of the steroid skeleton the isomerization of the Δ^8(9)^ double bond of 4α-methylergosta-8,24-dien-3β-ol (**7**) into a Δ^7(8)^ double bond of stigmasta-7,24-dien-3β-ol (avenasterol) (**8**), the desaturation at C-5 of the latter to produce the 5,7-dienic system of stigmasta-5,7-dien-3β-ol (7-dehydrositosterol, **9**), and the reduction of the Δ^7(8)^ double bond to yield sitosterol (**10**), the major plant Δ^5^-sterol. Dashed arrows represent more than one enzymatic steps. I, isomerization by a Δ^8^-Δ^7^-sterol isomerase (SI/HYD1, At1g20050), C-4 demethylation by a complex formed by a 4α-methyl-Δ^7^-sterol-4α-methyloxidase (SMO2, At1g07420, At2g29390), a 3β-hydroxysteroid dehydrogenase/C4-decarboxylase (βHSD, At2g33630), a sterone ketoreductase (SR, At5g18210) and the complex anchor ERG28 (At1g10030)^33,34^, and side chain alkylation by a 24-methylene lophenol-C28-methyltransferase (SMT2, At1g20330, At1g76090); II, desaturation by a Δ^7^-sterol-C5(6)-desaturase (C5-DES/DWARF7/STE1, At3g02580, At3g02590) and finally side chain reduction at C-24 by a Δ^5^-sterol-Δ^24^-reductase/isomerase (DIM/DWARF1, At3g19820). The complete sitosterol biosynthetic pathway is given in Supplementary Fig. 1. (**C**) *Arabidopsis thaliana* wild-type and *dwarf 5* plants. Steroid nomenclature refers to the International Union of Pure and Applied Chemistry (IUPAC) on the nomenclature of steroids (http://www.sbcs.qmul.ac.uk/iupac/steroid/)^35^. Sterol mass spectra produced in the present work were assigned based on available records^36^.

Vitamin D is a lipid critically important for the development of a healthy skeleton in vertebrates and for the maintenance of calcium and phosphorous homeostasis^4^. The identification of vitamin D was achieved in the twenties and thirties during the course of studies designed to elucidate the structure of an antirachitic factor present in the food or in UV-B irradiated skin^5^. Structurally the molecules collectively called vitamin D (D_2_ to D_6_) are secosteroids produced by the photoconversion of dienic Δ^5,7^-sterols upon UV-B irradiation^6^. The most popular of these compounds are vitamin D_2_ (cholecalciferol) and vitamin D_3_ (ergocalciferol), of animal and fungal origins, respectively (Fig. 1A). The photochemistry and synthetic chemistry of vitamin D are described^6^ however the supply of this important dietary compound for human or livestock consumption mostly relies on animal products such as cod liver oil. Vitamin D deficiency is caused by the lack of solar radiations in the UV-B wavelength (strictly dependent on latitude and climate), which abolishes the photoconversion of Δ^5,7^-cholesterol into vitamin D_3_ in the skin. Vitamin D deficiency is still a major health problem, it reduces immune defence and increases the risk of developing osteoporosis, cancer, cardiovascular diseases and diabetes^7,8^. Described as a pluripotent steroid hormone, vitamin D_3_ is transported from the skin to the plasma and is subsequently enzymatically oxidized in liver and kidney into the active form 1-hydroxyvitamin D_3_ and 1α,25-dihydroxyvitamin D_3_ (Fig. 1A).

Vitamin D_3_ and its oxidized derivatives were detected in a few plant species however the general occurrence of vitamin D in plants has received little attention^9–11^. Species of the *Solanum* or *Nicotiana* genera (such as tomato and tobacco) attracted particular attention because they contain cholesterol in significant amount: about 10% or more of the total sterols compared to 1–2% in other families^12^. In these plants, sterol biogenesis displays a cholesterol-specific biosynthetic segment that parallels the biosynthesis of 24-methylsterols (campesterol, brassicasterol) and 24-ethylsterols (sitosterol, stigmasterol)^13^. Vitamin D_5_ (sitocalciferol) (Fig. 1A) is a synthetic analog of vitamin D currently described in data bases (e.g. Human Metabolome Data Base, http://www.hmdb.ca/metabolites/HMDB0040586) but not reported in plant metabolomes although its precursor Δ^5,7^-sitosterol (also called 7-dehydrositosterol, 7-DHS) is a natural biosynthetic intermediate of sitosterol, the latter being one of the most abundant lipids on earth.

## Results

**Figure 2:**
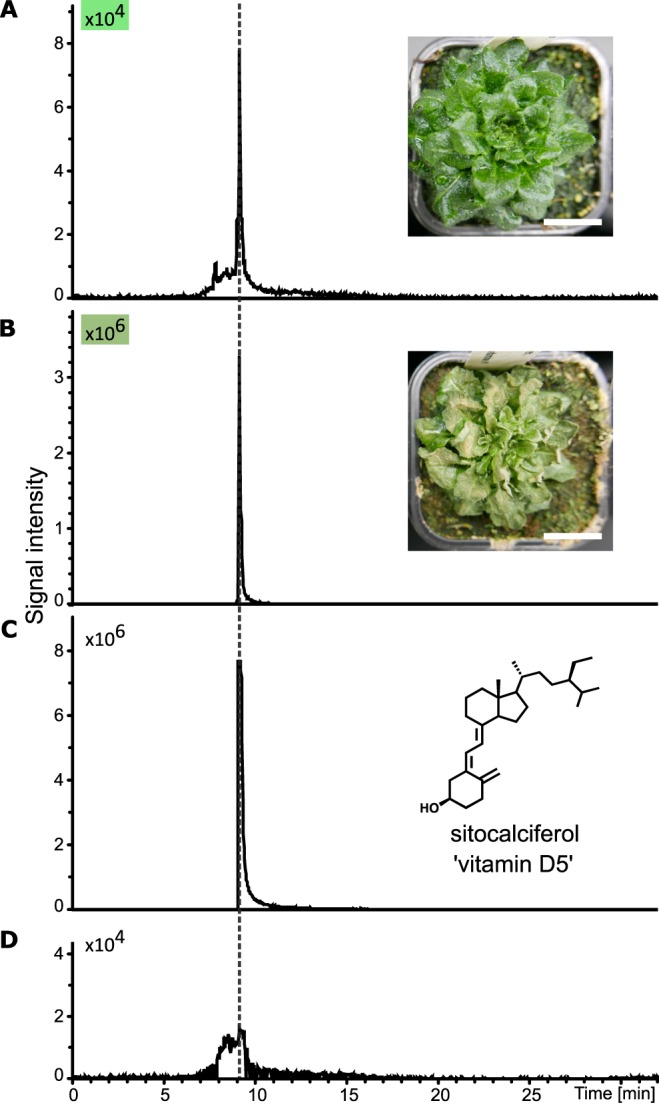
UHPLC- HR-MS/MS analysis of vitamin D_5_. Extracted ion chromatograms (EIC) of C_29_H_48_O [M + H] + (m/z 413,3778 +/− 0.01) in *dwarf 5-3* untreated plants (**A**), *dwarf 5-3* plants exposed to UV-B (**B**), vitamin D_5_ (sitocalciferol) standard (**C**) and mock extract (**D**). Retention time is represented as a dashed line. Scale bars represent 20 mm.

**Figure 3:**
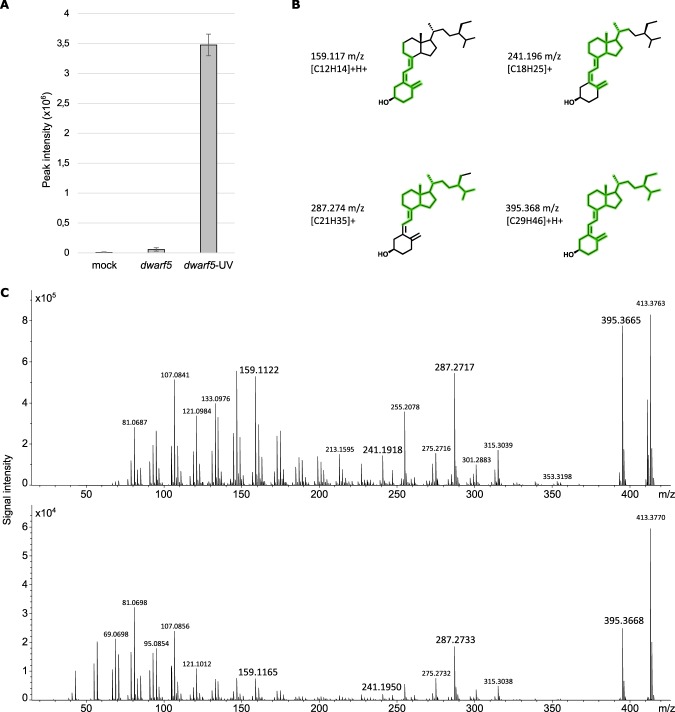
Mass spectral characterization of vitamin D_5_. Peak intensity of vitamin D_5_ was approximately 50 times higher in *dwarf 5-3* plants exposed to UV-B (**A**) than in non-exposed plants. Vitamin D_5_ was identified by interrogation of MetFrag, which displayed 49 fragments covering the entire skeleton of the molecule. The four major fragments showing the coverage of the molecule are highlighted in green in (**B**), other fragments are described in Table S1. Identification was confirmed by comparing the MS/MS spectra obtained from the samples (**C**, upper part) and from the standard (**C**, lower part).

Sitosterol biosynthesis requires the mandatory reduction of the C-7(8) double bond of Δ^5,7^-sitosterol by Δ^5,7^-sterol-Δ^7^-reductase (Figs 1B and S1) exactly like the conversion of Δ^5,7^-cholesterol to cholesterol^1,14,15^. The photoconversion of 7-DHS to vitamin D_5_ is therefore likely to occur but this has never been demonstrated *in planta* after exposure to artificial UV-B light. To achieve this, we cultivated an *Arabidopsis thaliana* mutant line *dwarf5* carrying the *dwarf5–3* allele of the gene encoding the Δ^5,7^-sterol-Δ^7^-reductase enzyme (called DWARF5) and exposed it to UV-B light. The *dwarf5–3* missense mutation (R400Z) causes the accumulation of Δ^5,7^-sterols, particularly 7-DHS, and a concomitant depletion in pathway end-products campesterol and sitosterol predominantly found in the wild-type^16,17^. Consequently, *dwarf5* mutant plants are expected to contain detectable amounts of vitamin D_5_ due to the high level of 7-DHS that can undergo photolysis. The *dwarf5* plants are characterized by short height, dark green round leaves, and a slow growth rate caused by insufficient levels of campesterol-derived brassinosteroids (Fig. 1C)^16^. UV-B light produced a dramatic effect on plant tissue integrity however compatible with extractive chemistry and analytical biochemistry (Fig. 2). A pure standard of synthetic vitamin D_5_ (Fig. S2**)** was used to implement an LC-MS preparative and analytical pipeline (Fig. S3). The workflow included first a pre-purification step on TLC (Fig. S4) followed by a semi-preparative HPLC-MS step, this to avoid the risk of interferences with other compounds especially heavy loads of leaf pigments. High resolution quadrupole time of flight (QTOF) tandem mass spectrometry coupled to atmospheric pressure photoionization (APPI) of compounds was implemented in this work to startedly point out the presence of vitamin D_5_ in *Arabidopsis thaliana dwarf5* leaf material, both in untreated (Fig. 2A) and UV-B-treated rosettes (Fig. 2B). In the latter, the peak area at m/z [M + H^+^] 413.3778 was about fifty times more intense than in the former (Fig. 3A). Mass spectral signatures unambiguously identified vitamin D_5_ i.e. 9,10-secostigmasta-5,7,10(19)-trien-3β-ol (Fig. 3B,C, Table S1). Wild-type plants were also submitted to the UV-B light treatment. The analysis of extracts from control and UV-B wild-type treated rosettes revealed a lack of detectable peaks of vitamin D_5_ (Fig. S5). The overaccumulation of vitamin D_5_ in *dwarf5* plants after UV-B light exposure is in agreement with a decrease of its precursor 7-DHS (Table 1). In fact, sterol profiling clearly showed a dramatic reduction in the amount of 7-DHS concomitantly to UV-B exposure, decreasing from 42% of the total sterols to 25% and 14% after 4 and 7 days, respectively, of the treatment (Table 1). Likewise, the proportion of other sterols remained almost unchanged between irradiated and non-irradiated *dwarf5* plants (Table 1). The accurate quantification of vitamin D_5_ production and 7-DHS consumption was not readily achievable since the methods implemented to extract and measure sterols on the one hand (GC-FID, GC-MS) and vitamin D_5_ on the other hand (QTOF) are differing by their sensitivity and resolution. Wild-type plants exhibited a strong increase of stigmasterol at the expense of sitosterol upon UV-B exposure (Table 1). The latter is desaturated at C-22 on the side chain by the enzyme CYP710A1 to produce stigmasterol^18^. Several biotic and abiotic factors acting on the expression of CYP710A1 are known, including pathogenic interactions, reactive oxygen species, and UV-B light^19,20^. UV-B–mediated 7-DHS decay in *dwarf5* plants may be explained by its photoconversion into vitamin D_5_ similarly to the conversion of ergosterol and Δ^5,7^-cholesterol into vitamin D_2_ and vitamin D_3_, respectively^21^.

**Table 1:** Sterol composition of leaf material untreated or exposed to UV-B irradiation.

	wild-type			*dwarf5-3*			
	t 0 d	t 7 d	t 7 d + UV	t 0 d	t 7 d	t 4 d + UV	t 7 d + UV
% sterol*							
cholesterol	3.8	2.3	3.5	1.0	1.0	3.0	nd
Δ^7^-cholesterol	2.3	2.4	3.3	3.8	1.0	4.7	10.0
brassicasterol	1.4	1.1	1.1	nd	nd	nd	nd
Δ^5,7^-cholesterol	nd	nd	nd	nd	nd	4.5	10.7
campesterol	16.2	13.6	15.0	3.8	4.0	2.7	tr
Δ^7^-campesterol	nd	nd	nd	4.1	5.5	3.3	8.3
stigmasterol	**4**.**2**	**4**.**0**	**30**.**2**	tr	nd	5.2	nd
Δ^5,7^-campesterol	nd	nd	nd	nd	nd	7.3	nd
sitosterol	**67**.**2**	**73**.**2**	**45**.**9**	8.8	6.2	8.1	15.8
isofucosterol	4.9	3.3	1.0	8.5	5.7	8.6	9.5
Δ^8^-sitosterol	nd	nd	nd	8.5	10.5	5.5	8.0
Δ^5,7^-sitosterol	nd	nd	nd	**42**.**3**	**45**.**3**	**25**.**7**	**14**.**9**
Δ^7^-sitosterol	nd	nd	nd	19.2	20.8	13.3	22.8
24-methylenelophenol	nd	nd	nd	nd	nd	8.1	nd
cycloartenol	nd	nd	nd	tr	tr	tr	nd
% total	100	100	100	100	100	100	100
total amount in mg.g-1 dry weight							
sample A	1.31	1.28	1.52	0.31	0.420	0.32	0.19
sample B		1.06	1.13	0.49		0.19	0.15
sample C		1.26					

*Percent of the total GC-FID peak areas as mean values of 2 or 3 data points, to the closest decimal. t, time; d, days; nd, not detected.

## Discussion

There are several outputs of the detection of vitamin D_5_ in the model plant *Arabidopsis thaliana*. The presence of vitamin D in plants is not anymore restricted to high-cholesterol containing plant species that produce some amounts of cholestane-derived vitamin D_3_. To the most abundant plant sterol backbone, namely, the stigmastane type, corresponds a secosteroid resulting in the present study from a UV-B mediated photochemical reaction. This work will certainly allow a further survey of vitamin D distribution in plant species and a refined profiling, quantification, and mapping of vitamin D. Most strikingly, the detection of vitamin D_5_ in *dwarf5* in the absence of high UV-B light exposure suggests an effect of a small light exposure (presumably due to the emission spectrum of white light fluorescent tubes), or more speculatively the existence of a non-photochemical formation of secosteroids in *Arabidopsis thaliana*. In fact, alternative enzymatic reactions converting dienic sterols to vitamin D have been hypothesized in an attempt to explain the abundance of vitamin D in animals living out of sunlight reach such as deepsea fishes or subterranean mammals^22^. Likewise, the abundance of Δ^5,7^-cholesterol, vitamin D_3_, 25-hydroxyvitamin D_3_ and 1,25-dihydroxyvitamin D_3_ in *Solanum glaucophyllum* (also called *Solanum malacoxylon*) may support to some extent the hypothesis of a non-photochemical production of vitamin D derivatives^23^. It is worth noting that *Solanum glaucophyllum*, a nightshade endemic to South America, is considered responsible for cattle hypervitaminosis after its overconsumption in grazing pastures in the Pampas region.

At present, it is not known whether vitamin D_5_ and possible derivatives or conjugates play a biological role in plant development similarly to the action of vitamin D_3_ in humans, and this at physiological levels of solar UV-B light, which remains to be tested. In plants, UV-B are perceived by the photoreceptor UVR8 (UV resistance locus 8)^24^. In the nucleus, the UVR8 active monomer/inactive dimer photocycle acts in interaction with the ubiquitin ligase COP1 (constitutive photomorphogenesis 1) on downstream regulators like the transcription factor HY5 (elongated hypocotyl 5) implied in photomorphogenesis and stress acclimation^25^. The putative link between the UVR8 sensor and a steroid status of the plant cell remains to be examined. UV-B induce the biosynthesis of phenylpropanoids^26^, one of the largest and oldest plant metabolome, suggesting indeed that light may also act as a master regulator of unexplored aspects of plant metabolism^27^.

Vitamin D_3_ hydroxylated derivatives are mostly investigated as drugs for cancer prevention and treatment due to their antiproliferative action. However, the amount of 1,25-dihydroxyvitamin D_3_ (calcitriol) needed to suppress the growth of fast proliferating cells also induced a toxic vitaminose because of the high calcemic property of the compound that was causing calcinosis of soft tissues and organs and eventually the death of patients^28^. One of the most extensively studied antiproliferative analogs of calcitriol is 1α-hydroxyvitamin D_5_ because this compound is fourfold less calcemic than 1,25-dihydroxyvitamin D_3_ and exhibits a similar biological activity^28,29^. Antiproliferative properties of synthetic 1α-hydroxyvitamin D_5_ was observed on models for breast, prostate-, and colon- cancer, except melanoma-cancer. So far, the use of vitamin D_5_ in experimental designs or for human daily consumption has been restricted due to the difficulty in sampling the compound from natural sources or to synthesize it chemically^29^. Artificial UV-B irradiation is a well-established industrial process to increase the level of vitamin D_2_ in mushrooms (*i*.*e*. conversion of ergosterol to ergocalciferol) or of vitamin D_3_ in cholesterol-rich plants (*i*.*e*. 7-DHC to cholecalciferol)^21^. The clear-cut identification of vitamin D_5_ in *Arabidopsis thaliana dwarf5* genotype opens new avenues to develop a cell technology approach based on the *dwarf5* mutant as a biotechnological tool for vitamin D_5_ production.

Standard and accurate methods to analyze vitamin D and its active hydroxylated forms are required both in the context of biotechnology initiatives but also in public health policies. Liquid chromatography coupled to electrospray ionization and high-resolution tandem mass spectrometry (LC-HR-MS/MS) recently improved the quantification of 25-hydroxyvitamins in the frame of official guidelines on bioanalytical method validations^30^. The method set up in this work to detect vitamin D_5_ stands out thanks to the development of atmospheric pressure photoionization (APPI) as a method of choice for high efficiency ionization of hydrophobic small molecules and on high resolution spectrometers able to determine accurate m/z values for molecular species and their pattern of fragmentation.

## Materials and Methods

### Plant material

*Arabidopsis thaliana* (L.) plants from the ecotypes Enkheim 2 (En-2) wild type and mutant *dwarf5–3* were obtained from the Nottingham Arabidopsis Stock Center NASC seed stock N1138 and N402^16,17^. Plants were grown in a controlled chamber with a light/dark regime of 12 hours white light (Lumilux T5 HE fluorescent tubes, Osram) and 12 hours dark, and a temperature setting of 22 °C during the light period and 19 °C during the dark period. Four weeks old seedlings were transferred to pots (7 cm diameter) and grown to maturity. Plants at the fully expanded rosette stage (8 to 12 weeks) were exposed to short repetitive sequences of UV-B light in addition to normal light supplied according to the light/dark cycle. Rosettes were exposed to UV-B light for 5 minutes within every hour for 7 days. UV-B light was generated by a UVP-302-15 lamp equipped with two 15 W tubes emitting at a wavelength of 302 nm. The intensity of the emission was measured with a photometer (model ILT1400BL, equipped with SEL005 detector and TLS312 filter, International Light Technologies, Denmark). An irradiance of 280 µJ/sec/cm^2^ was detected at a distance of 80 cm from the UV-B lamp, corresponding to the top of the pots. For comparison, the solar UV-B average irradiance on earth is 8 µJ/sec/cm^2^ ^31^. The same lamp was used for all experiments performed in this study. Series of 20 plants (4 rows of 5 pots) were placed under the UV lamp to perform the treatment. Control plants were kept in the growth chamber without additional exposure to artificial UV-B light. At the end of the treatment, whole rosettes were collected in liquid nitrogen and freeze-dried in a lyophilisator (ScanVac model CoolSafe 100–4 or Heto Drywinner, ThermoFisher). Samples were stored at −20 °C under argon atmosphere until analysis.

### Authentic chemical standards

Authentic vitamin D_5_^32^ ((5Z,7E)-9,10-secostigmasta-5,7,10(19)-trien-3β-ol, CAS 71761-06-3, 85% chemical purity) was purchased from BOC Sciences, Shirley, NY 11967, USA. Sitosterol (Purity ≥ 95%) and 7-dehydrocholesterol (7-DHC) (Purity > 98%), were purchased from Sigma-Aldrich (Steinheim, Germany) and used as standards. Stock solutions of the standards were prepared in benzene (Fluka) and stored at −20 °C.

### NMR analysis

NMR spectra were recorded in CDCl3 with a BRUKER Advance II instrument equipped with a ^1^H/^13^C 5 mm cryoprobe (DCH ^13^C/^1^H/D z-grad). ^1^H spectra were taken at 500 MHz and ^13^C spectra at 125 MHz using a distortionless enhancement by polarization transfer (DEPT) pulse sequence.

### Extraction of vitamin D_5_

Lyophilized rosettes (10–15 grams dry weight) were transferred into a 1 L Erlenmeyer flask and homogenized in 250 mL methanol with 6% (w/v) KOH using an Ultra Turrax^®^. In order to prevent loss of vitamin D compounds due to thermal isomerisation and oxidation, the saponification was carried out overnight (approx. 18 hours) at 28°C in a rotating incubator at 180 rpm. Subsequently 0.5 volume of water then 1 volume of a mixture of chloroform:pentane (1:4, v/v) were added to the methanolic fraction to perform a biphasic extraction of the unsaponifiable in a 1 L separatory funnel. Organic fractions were recovered by centrifugation at 4000 g for 5 minutes (Falcon tubes) or by decantation (separatory funnel) at room temperature under dim light. Extractions were done three times. The organic fractions were combined then evaporated to dryness in a rotary evaporator at 30 °C (Rotavapor^®^ model R-215, Büchi, Switzerland) under dim light. The dry residues were resuspended in chloroform:pentane (1:4, v/v), transferred to amber glass vials, dried under a stream of Nitrogen gas and stored in Argon atmosphere at −20 °C prior to analysis. Chloroform, methanol and pentane were GC grade (Fluka or Carlo Erba). Potassium hydroxide pellets (for analysis) were from Merck (Damstadt, Germany).

### Preparation of vitamin D_5_-enriched fractions

The unsaponifiable plant extracts were resolved on TLC plates (Merck 60 F254 silica gel, 20 × 20 cm × 0.25 mm) using a mixture of dichloromethane:ethyl acetate (9:1, v/v) (two runs). Authentic vitamin D_5_, 7-DHS and 7-DHC were spotted on the plates as references. Developed plates were exposed to UV light at λ = 254 nm to reveal dienic steroids by their strong purple fluorescence. The following R_f_ were recorded for standards used in this study: vitamin D_5_ (R_f_ = 0.68), 7-DHS (purified from Arabidopsis thaliana *dwarf5* plants) (R_f_ = 0.56) and 7-DHC (Sigma) (R_f_ = 0.56). A fraction (R_f_ = 0.62 to R_f_ = 0.68) containing compounds of the same mobility as vitamin D_5_ was scrapped off the plate and eluted with chloroform:pentane (1:4, v/v), transferred to amber glass vials, dried under a stream of nitrogen gas and stored in Argon atmosphere at −20 °C prior to HPLC purification and mass spectrometry analysis. An aliquote of the fraction was derivatized as described in above and analysed by GC-MS to verify the absence of 7-DHS, the precursor of vitamin D_5_.

### Purification of vitamin D_5_

Fractions prepared by TLC were chromatographed using a semi-preparative HPLC-C8-ESI-MS protocol coupled to a fraction collector. This process was performed on a HPLC Waters 2695 XE Separations Module (Waters, Mildorf, MA, USA) coupled to a Waters 2998 Photodiode Array Detector PAD, a Waters Acquity QDA mass spectrometry detector and a Waters Fraction Collector III. Chromatographic separation was achieved using an XBridge® Prep C8 column (10 × 250 mm, 5 µm; Waters), coupled to an XBridge® Prep C8 pre-column (10 × 10 mm, 5 µm; Waters). The mobile phases selected were 75% methanol/in water with 0.01% formic acid (solvent A) and 99.99% isopropanol with 0.01% formic acid (solvent B). The separation started with 100% A maintained for 10 min; followed by 30 min gradient to reach 50% A. It was followed by a gradient to reach 100% B in 10 min, maintained for 10 min. Then 5 min gradient was applied to reach 100% of solvent A, and maintained for 15 min. Total run time was 80 min. The column was operated at 47 °C with a flow-rate of 3 mL.min^−1^, the sample injection volume was 100 µL. The parameters involving the MS detection and ESI ionization were as follow: the ESI probe temperature was set to 600 °C, and the source temperature to 120 °C. The cone voltage was set to 10 V and the capillary voltage to 0.8 V. The ionization mode was positive. The selected ion recording (SIR) MS mode was used to detect vitamin D_5_ at m/z 413. Automatic start collection of vitamin D_5_ was set at 37.50 min during 90 sec. Data acquisition and analyses were performed with the MassLynx software (ver.4.1) running under Windows 7 professional on a Pentium PC. Collection tubes were placed on ice in the darkness to avoid photo-degradation. All fractions were combined and dried under vacuum for LC-HR-MS/MS analysis.

### Analysis of vitamin D_5_

Chromatography and mass spectrometry of vitamin D_5_ were performed on the UltiMate 3000 UHPLC system (Thermo) coupled to the ImpactII (Bruker) high resolution Quadrupole Time-of-Flight (QTOF) equipped with an atmospheric pressure photoionization (APPI) source. Chromatographic separation was achieved on an Acquity UPLC® BEH C_8_ column (2.1 × 100 mm, 1.7 µm, Waters) coupled to an Acquity UPLC BEH C_8_ pre-column (2.1 × 5 mm, 1.7 µm, Waters). Injection was performed in a full loop mode with 10 µL of the samples, then subjected to a gradient of solvent A (25% H_2_O/75% MeOH/0.01% formic acid) and B (100% isopropanol/0.01% formic acid). Chromatography was carried out at a flux of 0.380 mL.min^−1^ with 100% solvent A for 5 minutes, and then reached 50% B at 0.350 ml.min^−1^ at 8 minutes for a plateau of 4 minutes. At 12 minutes, the flux was set at 0.250 ml.min^−1^ and a ramp was used to get to 100% solvent B at 17 min, kept until 27 min. Then, solvent B was brought back to 50% at 0.350 mL.min^−1^, kept at 50% from 29 to 34 min, and then 100% A was achieved at 40 min. Total run time was 45 minutes to stabilize the chromatographic conditions at 0.250 mL.min^−1^ and 100% solvent A. The column was operated at 47 °C. Nitrogen was generated from pressurized air by Nitro 35 Nitrogen generator (Claind) and used as nebulizing gas (1.5 Bar) and dry gas (1.5 L.min^−1^). Capillary voltage was set at 700 V, dry temperature at 225 °C and vaporizer temperature at 300 °C. Samples were analysed in positive Auto MS/MS mode with ion energy set at 6 eV and collision cell energy at 20 eV in a first time and then 5 eV to implement the standard in spectral library. Calibration was set from 50 to 2200 daltons (Da) using the APCI/APPI tuning mix from Agilent (StdDev 0.51ppm) in quadratic calibration mode.

### Sterol analysis

Lyophilized plant tissues (50 mg dry weight) were homogenized in 15 mL methanol with 6% (w/v) KOH using an Ultra Turrax^®^. Subsequently 0.5 volume of water then 1 volume of hexane were added to the methanolic fraction to perform a biphasic extraction of the unsaponifiable. Dried extracts were acetylated in 100 μL toluene with 50 μL acetic anhydride and 50 μL pyridine at 70 °C for one hour. Sterol acetates were resolved as a single fraction at R_f_ = 0.5 on TLC plates (Merck 60 F254 silica gel) with one run in dichloromethane. The steryl acetate fractions were scrapped off the plate and recovered in dichloromethane then dried. Betulin diacetate (20 μL of a 1 mg/ml solution) was added as internal standard. Samples in 500 μL hexane were analysed by GC-MS and GC-FID for structural identification and quantification. GC-MS (Agilent 6890 GC System, Agilent 5973 Mass Selective Detector EI 70 eV, GC-MSD 5973 software) and GC-FID (Varian 8300, Varian Star software) were equipped with a HP-5MS and a DB5 glass capillary columns (wall coated, open, tubular; 30 m; 0.32 mm i.d., 0.25 mm; Agilent J&W, USA). The GC program included a fast increase from 60 °C to 220 °C (30 °C/min) and a slow increase from 230 °C to 280 °C (2 °C/min). The carrier gases were He 1 ml × min^−1^ for CG-MS and H_2_ (2 ml × min^−1^) for CG-FID.

## Electronic supplementary material

Supplementary figures and tables.

## Data Availability

Data generated in the experiments described in this article are available upon reasonable request.

## References

[1] Schaller Hubert (2010). Sterol and Steroid Biosynthesis and Metabolism in Plants and Microorganisms. Comprehensive Natural Products II.

[2] Grove MD (1979). Brassinolide, a plant growth-promoting steroid isolated from *Brassica napus* pollen. Nature.

[3] Li J, Nagpal P, Vitart V, McMorris TC, Chory J (1996). A role for brassinosteroids in light-dependent development of Arabidopsis. Science.

[4] Dixon KM, Mason RS (2009). Vitamin D. Int. J. Biochem. Cell. Biol..

[5] Wolf G (2004). The discovery of vitamin D: the contribution of Adolf Windaus. J. Nutr..

[6] Zhu GD, Okamura W (1995). Synthesis of vitamin D. Chem. Rev..

[7] Gorham ED (2005). Vitamin D and prevention of colorectal cancer. J. Steroid Biochem. Mol. Biol..

[8] Holick MF (2008). The vitamin D deficiency pandemic and consequences for nonskeletal health: Mechanisms of action. Molecular Aspects of Medicine.

[9] Prema TP, Raghuramulu N (1996). Vitamin D-3 and its metabolites in the tomato plant. Phytochemistry.

[10] Aburjai T, Al-Khalil SA, Abuirjeie M (1998). Vitamin D-3 and its metabolites in tomato, potato, eggplant and zucchini leaves. Phytochemistry.

[11] Boland R, Skliar M, Curino A, Milanesi L (2003). Vitamin D compounds in plants. Plant Science.

[12] Moreau RA, Whitaker BD, Hicks KB (2002). Phytosterols, phytostanols, and their conjugates in foods: structural diversity, quantitative analysis, and health-promoting uses. Prog. Lipid Res..

[13] Sonawane PD (2016). Plant cholesterol biosynthetic pathway overlaps with phytosterol metabolism. Nat Plants.

[14] Benveniste P (2004). Biosynthesis and accumulation of sterols. Annu. Rev. Plant Biol..

[15] Nes WD (2011). Biosynthesis of cholesterol and other sterols. Chem. Rev..

[16] Choe S (2000). Lesions in the sterol Delta (7) reductase gene of Arabidopsis cause dwarfism due to a block in brassinosteroid biosynthesis. Plant J..

[17] Schaller H (2003). The role of sterols in plant growth and development. Prog. Lipid Res..

[18] Morikawa T (2006). Cytochrome P450 CYP710A encodes the sterol C-22 desaturase in Arabidopsis and tomato. Plant Cell.

[19] Griebel T, Zeier J (2010). A role for beta-sitosterol to stigmasterol conversion in plant-pathogen interactions. Plant J..

[20] Winter Debbie, Vinegar Ben, Nahal Hardeep, Ammar Ron, Wilson Greg V., Provart Nicholas J. (2007). An “Electronic Fluorescent Pictograph” Browser for Exploring and Analyzing Large-Scale Biological Data Sets. PLoS ONE.

[21] Jasinghe VJ, Perera CO (2006). Ultraviolet irradiation: the generator of Vitamin D-2 in edible mushrooms. Food Chem..

[22] Norman TC, Norman AW (1993). Consideration of chemical mechanisms for the nonphotochemical production of vitamin D_3_ in biological systems. Bioorg. Med. Chem. Lett..

[23] Curino A, Skliar M, Boland R (1998). Identification of 7-dehydrocholesterol, vitamin D-3, 25(OH)-vitamin D-3 and 1,25(OH)(2)-vitamin D-3 in Solanum glaucophyllum cultures grown in absence of light. Biochim. Biophys. Acta.

[24] Wu D (2012). Structural basis of ultraviolet-B perception by UVR8. Nature.

[25] Ulm R, Jenkins GI (2015). Q&A: How do plants sense and respond to UV-B radiation?. BMC Biol..

[26] Bieza K, Lois R (2001). An Arabidopsis mutant tolerant to lethal ultraviolet-B levels shows constitutively elevated accumulation of flavonoids and other phenolics. Plant Physiol.

[27] Lakshmanan M (2015). Unraveling the Light-Specific Metabolic and Regulatory Signatures of Rice through Combined in Silico Modeling and Multiomics Analysis. Plant Physiol..

[28] Mehta RG, Mehta RR (2002). Vitamin D and cancer. J. Nutr. Biochem..

[29] Moriarty, R. M. & Albinescu, D. Synthesis of 1alpha-hydroxyvitamin D5 using a modified two wavelength photolysis for vitamin D formation. *J*. *Org*. *Chem*. **70**, 7624-7628. 10.1021/jo050853f 16149791

[30] Matysik S, Liebisch G (2017). Quantification of steroid hormones in human serum by liquid chromatography-high resolution tandem mass spectrometry. J. Chromatogr. A.

[31] Dring MJ, Wagner A, Franklin LA, Kuhlenkamp R, Luning K (2001). Seasonal and diurnal variations in ultraviolet-B and ultraviolet-A irradiances at and below the sea surface at Helgoland (North Sea) over a 6-year period. Helgoland Marine Research.

[32] Napoli JL, Fivizzani MA, Schnoes HK, Deluca HF (1979). Synthesis of Vitamin-D5 - Its Biological-Activity Relative to Vitamin-D3 and Vitamin-D2. Arch. Biochem. Biophys..

[33] Mialoundama AS (2013). Arabidopsis ERG28 tethers the sterol C4-demethylation complex to prevent accumulation of a biosynthetic intermediate that interferes with polar auxin transport. Plant Cell.

[34] Darnet S, Rahier A (2004). Plant sterol biosynthesis: identification of two distinct families of sterol 4α-methyl oxidases. Biochem J..

[35] Moss GP (1989). The nomenclature of steroids. Pure Appl. Chem..

[36] Rahier Alain, Benveniste Pierre (1989). Mass Spectral Identification of Phytosterols. Analysis of Sterols and Other Biologically Significant Steroids.

