# Supplementary material for: Vitamin D5 in Arabidopsis thaliana
Source: Sci Rep. 2018 Nov 5;8:16348. doi: 10.1038/s41598-018-34775-z (PMC6218535; doi:10.1038/s41598-018-34775-z)
Supplement: Supplementary file 1 — Supplementary figures and tables. [file 41598_2018_34775_MOESM1_ESM.pdf]

Vitamin D_5 in *Arabidopsis thaliana*
Supplementary Information

Daniele Silvestro[1¶‡], Claire Villette[2‡], Julien Delecolle[2], Carl Erik Olsen[1], Mohammed Saddik Motawia[1], Philippe Geoffroy[3], Michel Miesch[3], Poul Erik Jensen[1], Dimitri Heintz[2], Hubert Schaller[2*]

Affiliations

[1]Copenhagen Plant Science Centre, Department of Plant and Environmental Sciences, University of Copenhagen, Thorvaldsensvej 40, DK-1871 Frederiksberg C, Copenhagen, Denmark.

[2]Institut de Biologie Moléculaire des Plantes du CNRS, Université de Strasbourg, 12 rue du Général Zimmer, F–67083 Strasbourg, France.

[3]Institut de Chimie, Université de Strasbourg, 1 rue Blaise Pascal, F-67008 Strasbourg, France

[¶] Present address: Carlsberg Research Laboratory, Copenhagen

[‡]These authors contributed equally

[*]Correspondence should be addressed to H.S. (hubert.schaller@ibmp-cnrs.unistra.fr)

Supplementary figures legends and tables.

Fig.S1. Plant sterol biosynthesis. The Arabidopsis sterol biosynthetic pathway starting from 2,3-oxidosqualene is shown. The first enzymatic reaction is the addition of a methyl group at C-24 of cycloartenol by SMT1, sterol-C24-methyltransferase. A small proportion of the pool of cycloartenol is not methylated by SMT1 however enzymes downstream to SMT1 act on cycloartenol and its downstream products (dashed arrow from cycloartenol to cholesterol) to produce cholesterol as a pathway end-product (*13*). The non-consecutive C-4 demethylation reactions C4DM1 and C4DM2 (C-4 demethylation 1 and 2) are performed by a protein complex tethered by ERG28 (*33*) and comprising a sterol methyl oxidase SMO1 or SMO2 in the case of C4DM1 and C4DM2, a 4-carboxysterol-3β-hydroxysteroid-C4-decarboxylase, and a 3-oxosteroid reductase (*34*). CPI, cyclopropyl isomerase; CYP51, obtusifoliol-14-demethylase; C-14-SR, sterol-Δ14-reductase; 8SI, Δ^8-sterol-Δ^8-Δ^7-isomerase; SMT2, sterol-C28-methyltransferase; C5-DES, Δ^7-sterol-C5-desaturase; C7-SR, $\Delta^{5,7}$-sterol-Δ^7-reductase; C24-SR, Δ^5-sterol-Δ^{24}-isomerase/reductase; CYP710A, sterol-C22-desaturase.

Fig.S2. NMR spectra of an authentic standard of vitamin D5. a, ^{1}H NMR (500 MHz, $CDCl_3$): δ: 0.52 (s, 2H); 3.95 (m, 1H); 4.82 (d, *J* = 2.3Hz, 1H); 5.05 (d, *J* = 2.3Hz, 1H); 6.01 (d, *J* = 11.1 Hz, 1H); 6.22 (d, *J* = 11.1 Hz) ppm; **b, ^{13}C NMR (125 MHz, $CDCl_3$)**: δ: 145.3, 142.4, 135.1, 122.8, 117.7, 122.6, 69.3, 56.7, 56.6, 46.08, 46.05, 40.8, 36.7, 35.2, 34.1, 32.1, 29.9, 29.3, 29.2, 27.8, 26.3, 23.8, 22.4, 20.5, 19.2, 19.1, 14.3, 12.2 ppm. Chemical shifts were determined with respect to the solvent residual peak.

Fig.S3. Vitamin D_5 identification workflow. The unsaponifiable leaf extracts were chromatographed on TLC plates (step 1). Pre-purified vitamin D_5 extracts and an authentic commercial standard of vitamin D_5 were sampled with a fraction collector on a semi-preparative

HPLC-MS (step 2). Standard vitamin D_5 enabled the methodological developments. The fractions were dried in a centrifugal vacuum dryer and analysed in UHPLC-HR-MS/MS (step 3).

Fig.S4. Preparation of vitamin D_5-enriched fractions by TLC. The picture shows a chromatography of the unsaponifiable *Arabidopsis thaliana dwarf 5* leaf extracts. Samples were resolved on a TLC plates (Merck 60 F254 silica gel, 20x20cm x 0.25mm) using a mixture of dichloromethane:ethyl acetate (9:1, v/v). Authentic 7-DHS and vitamin D_5 were spotted on the plates as references. Developed plates were exposed to UV light at λ=254 nm to reveal dienic steroids by their strong purple fluorescence. The following R_f were recorded for standards used in this study: vitamin D_5 (R_f=0.68), 7-DHS (purified from Arabidopsis thaliana *dwarf5* plants) (R_f= 0.56). A fraction (R_f=0.62 to R_f=0.68, visualized as white frames on the photography of the TLC plate) containing compounds of the same mobility as vitamin D_5 was scrapped off the plate and eluted (see Material and Methods section).

Fig.S5. High resolution mass spectrometry analysis of wild-type *Arabidopsis thaliana* leaf material for the detection of vitamin D_5. The analytical pipeline described in Fig.S3 was implemented without the semi-preparation purification step to check the presence of vitamin D_5 in control and UV-B treated batches of plants prepared in triplicate samples.

Table S1. *In silico* fragmentation obtained using MetFrag for the identification of vitamin D_5, corresponding to MS/MS fragments obtained in biological samples.

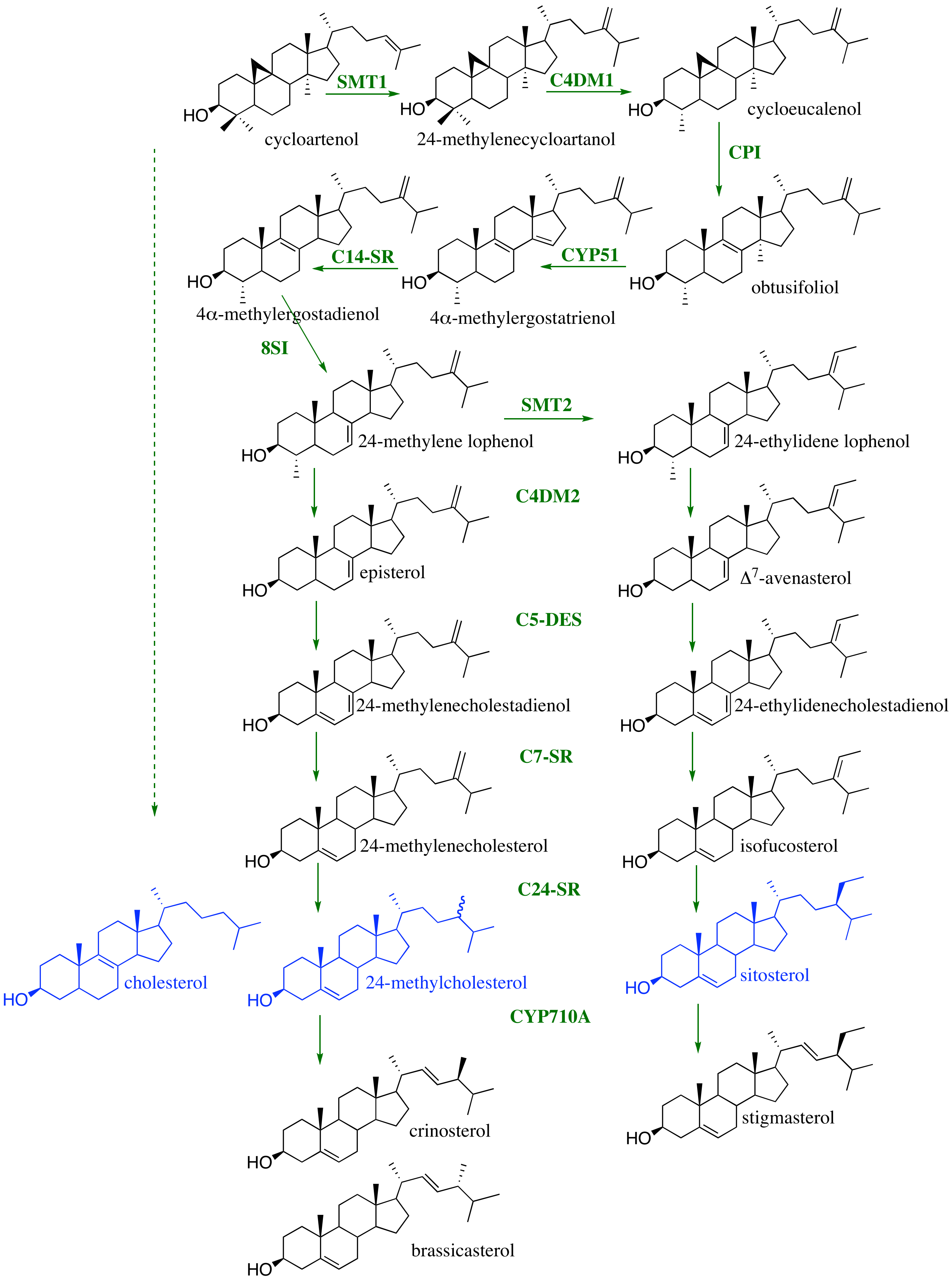

HO
cycloartenol
SMT1
24-methylenecycloartanol
C4DM1
cycloeucalenol
CPI
obtusifoliol
CYP51
4α-methylergostatrienol
C14-SR
4α-methylergostadienol
8SI
24-methylene lophenol
SMT2
24-ethylidene lophenol
C4DM2
episterol
Δ7-avenasterol
C5-DES
24-methylenecholestadienol
24-ethylidenecholestadienol
C7-SR
24-methylenecholesterol
isofucosterol
C24-SR
cholesterol
24-methylcholesterol
sitosterol
CYP710A
crinosterol
stigmasterol
brassicasterol

a. authentic vitamin D5 standard (1D 1H) CDCl3 500MHz

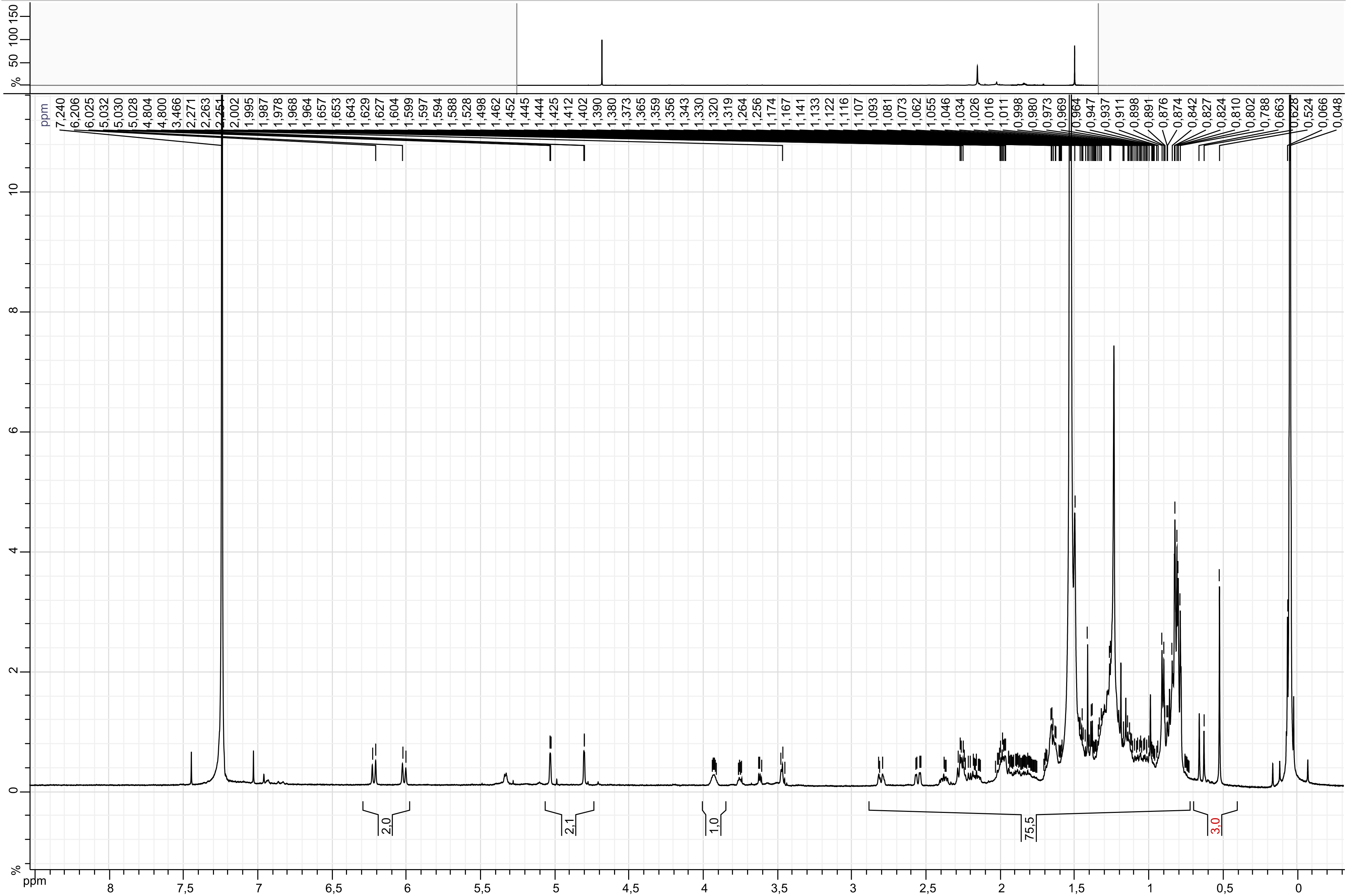

b. authentic vitamin D5 standard (1D 13C) CDCl3 500MHz

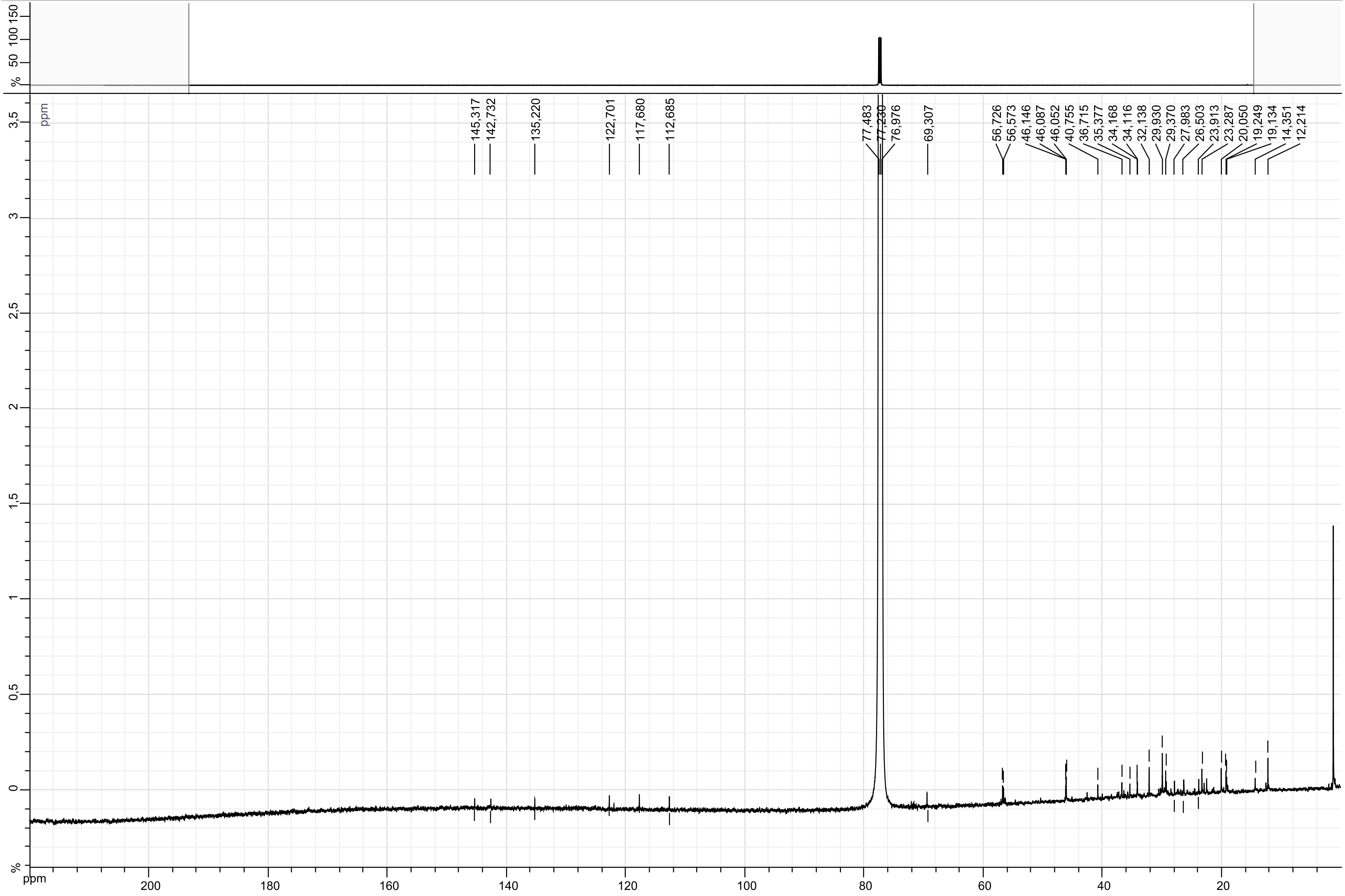

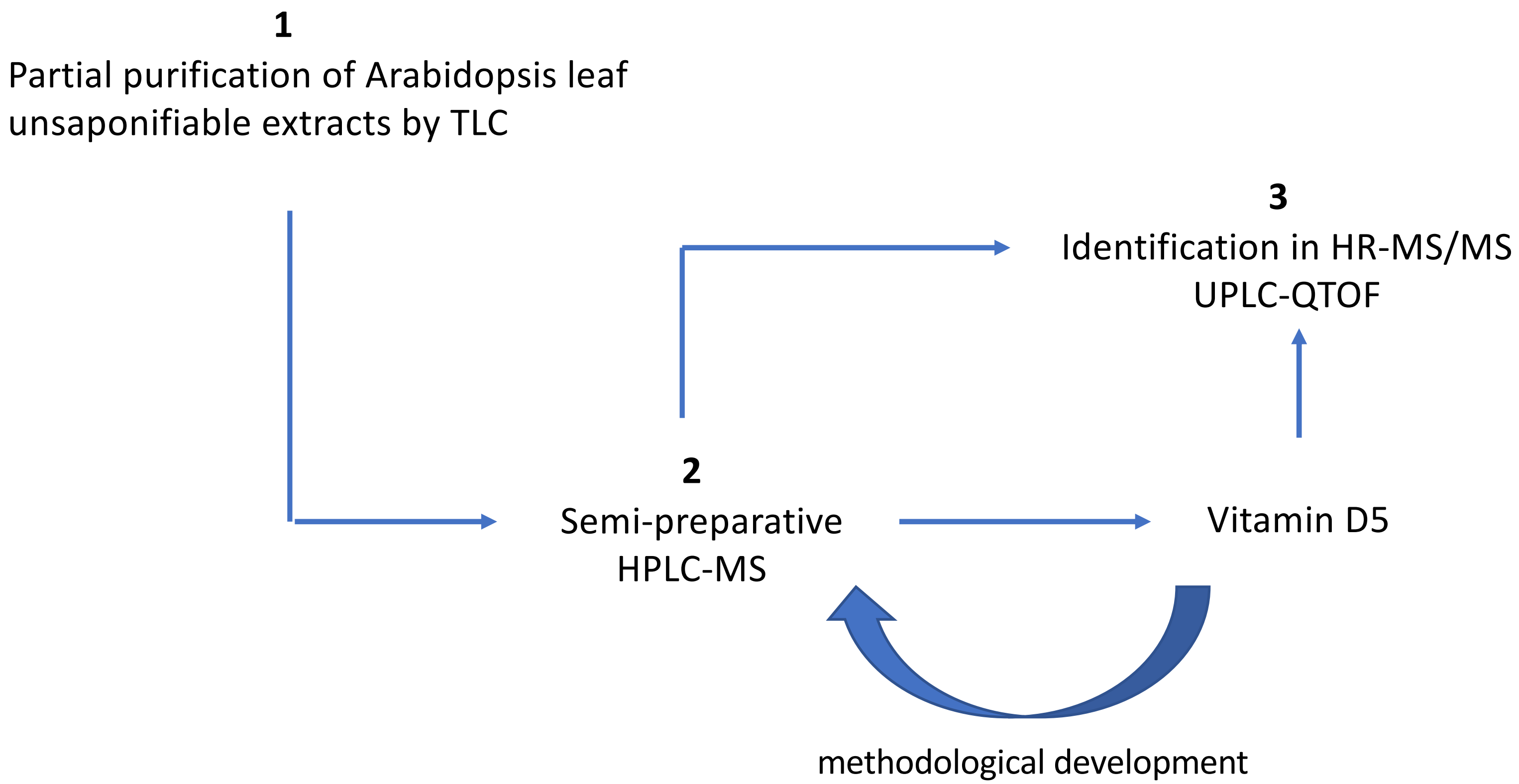
1
Partial purification of Arabidopsis leaf unsaponifiable extracts by TLC
3
Identification in HR-MS/MS
UPLC-QTOF
2
Semi-preparative
HPLC-MS
Vitamin D5
methodological development

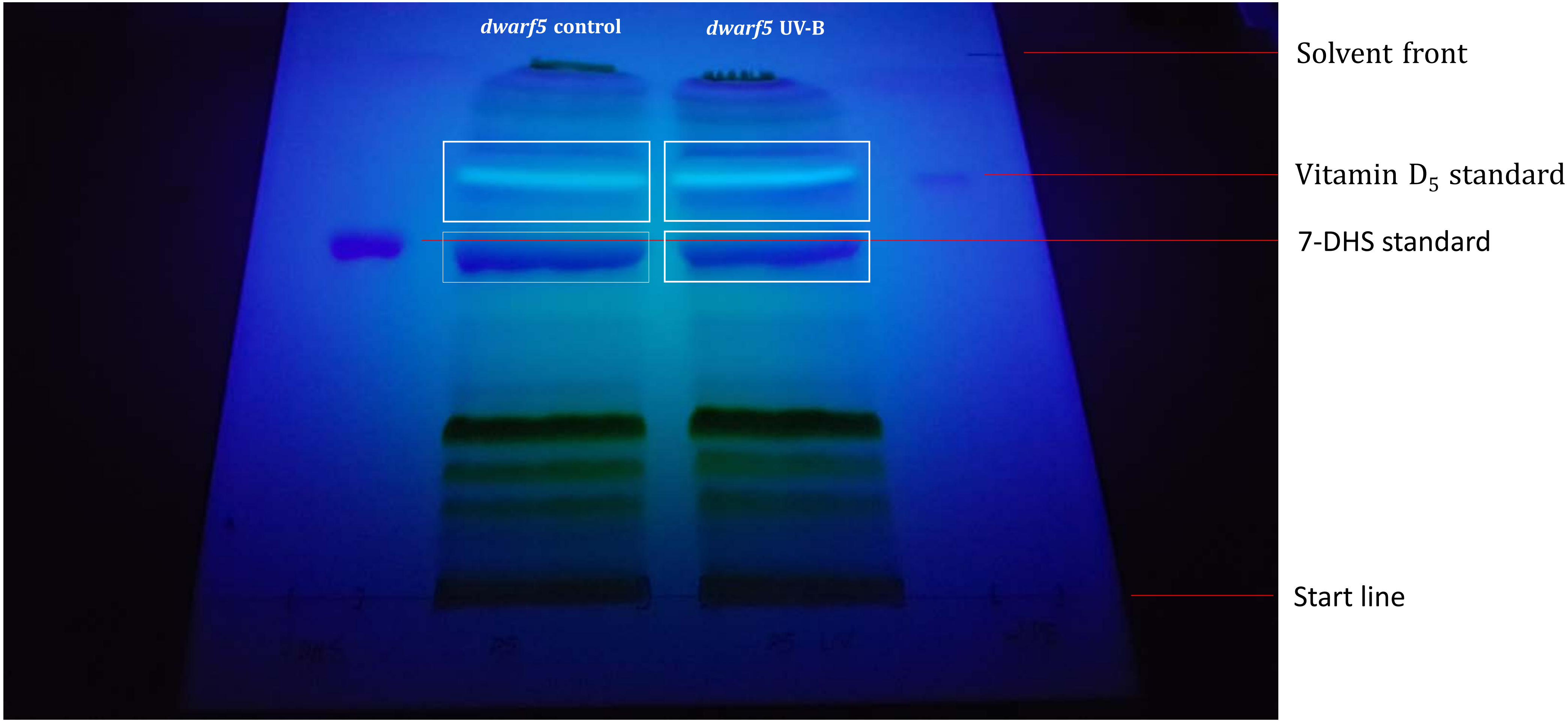

dwarf5 control
dwarf5 UV-B
Solvent front
Vitamin D_5 standard
7-DHS standard
Start line

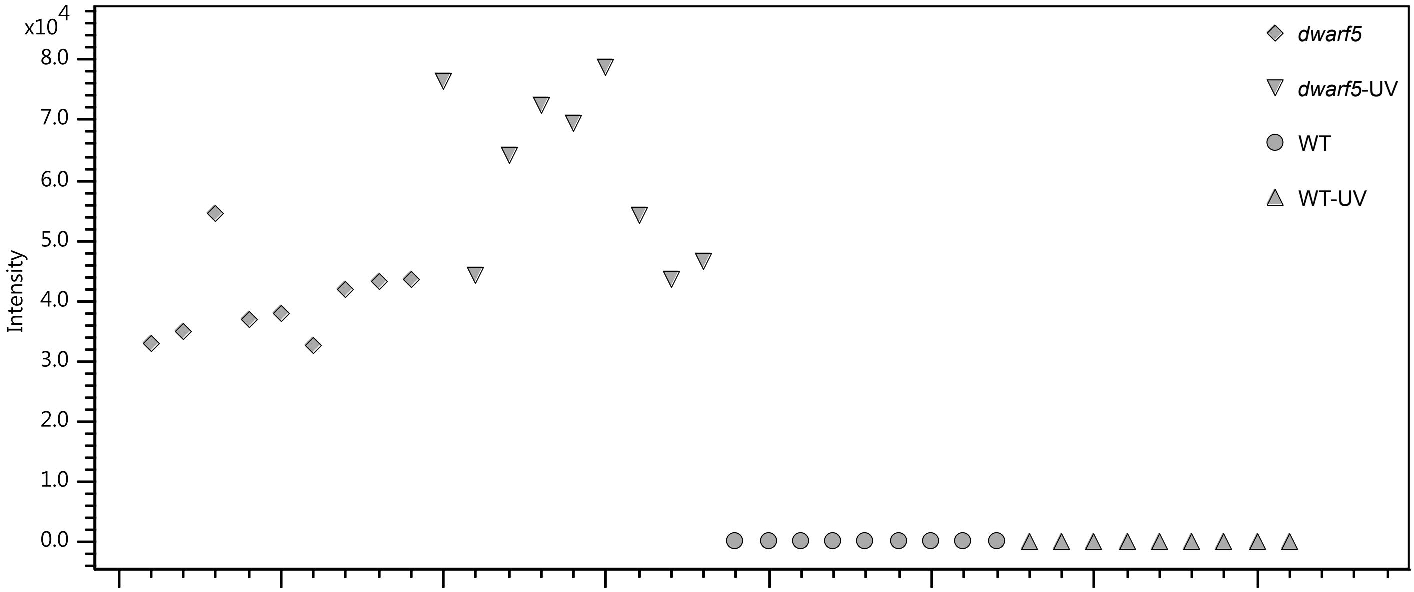
x10^4
8.0
7.0
6.0
5.0
4.0
3.0
2.0
1.0
0.0
Intensity
dwarf5
dwarf5-UV
WT
WT-UV

Supplementary Table S1. *In silico* fragmentation obtained using MetFrag for the identification of vitamin D_5, corresponding to MS/MS fragments obtained in biological samples.

Ion formula	m/z measured	Δ m/z (mDa)
[C6H9]+	81.070	0.2
[C6H12-H]+	83.086	[1H] 0.2
[C6H13]+	85.102	0.1
[C7H11]+	95.086	0.1
[C7H14-H]+	97.102	[1H] 0.1
[C8H9]+	105.070	0.2
[C8H9+H]+H+	107.086	[-2H] 0.1
[C8H13]+	109.102	0.1
[C8H16-H]+	111.117	[1H] 0.1
[C9H10-H]+	117.070	[1H] 0.1
[C9H10]+H+	119.086	[-1H] 0.2
[C9H13]+	121.102	0.1
[C9H15]+	123.117	0.1
[C10H10]+H+	131.086	[-1H] 0.3
[C10H15-2H]+	133.102	[2H] 0.1
[C10H15]+	135.117	0.1
[C10H17]+	137.133	0.3
[C11H12]+H+	145.102	[-1H] 0.2
[C11H16]+H+	149.133	[-1H] 0.2
[C12H14-H]+	157.101	[1H] 0.3
[C12H14]+H+	159.117	[-1H] 0.1
[C12H17]+	161.133	0.2
[C12H19]+	163.149	0.1
[C13H16-H]+	171.117	[1H] 0.3
[C13H17]+	173.133	0.1
[C12H16O]+H+	177.128	[-1H] -0.1
[C13H20-H]+	175.149	[1H] 0.1
[C13H20]+H+	177.164	[-1H] 0.1
[C14H19-2H]+	185.133	[2H] 0.2
[C14H19]+	187.148	0.3
[C14H22-H]+	189.164	[1H] 0.3
[C15H19]+	199.149	0.1
[C15H21]+	201.164	0.1
[C15H24-H]+	203.180	[1H] 0.1
[C16H21]+	213.164	0.0
[C16H23]+	215.180	0.1
[C17H23]+	227.180	0.1
[C18H25]+	241.196	-0.2
[C18H30]+H+	247.242	[-1H] 0.3
[C19H25+H]+H+	255.211	[-2H] 0.1
[C19H27O+H]+H+	273.222	[-2H] -0.2
[C20H34-H]+	273.258	[1H] -0.0
[C21H33]+	285.258	-0.0
[C21H35]+	287.274	-0.2
[C22H35]+	299.274	-0.2
[C22H38-H]+	301.290	[1H] -0.3

[C23H38]+H+	315.305	[-1H] -0.2
[C29H46-H]+	393.353	[1H] -0.5
[C29H46]+H+	395.368	[-1H] -0.3
